# Urea molasses mineral block under various feeding systems improved nutrient digestibility, productive performance and blood biochemical profile of Yaks

**DOI:** 10.1186/s12917-023-03676-3

**Published:** 2023-09-08

**Authors:** Muhammad Mobashar, Muhammad Tahir Khan, Maria Marjan, Shakoor Ahmad, Umar Farooq, Muhammad Farooq Khalid, Riaz Mustafa, Nadar Khan, Abu Bakkar Sadiq, Anwar Shah, Ahmed A. A. Abdel-Wareth

**Affiliations:** 1 Department of Animal Nutrition, Faculty of Animal Husbandry and Veterinary Sciences, The University of Agriculture Peshawar, Peshawar, 25130 Pakistan; 2 Khyber Pukhtunkhwa Wildlife Department Peshawar, Peshawar, Pakistan; 3 College of Veterinary Sciences, The University of Agriculture, Peshawar, 25130 Pakistan; 4 University of Agriculture Faisalabad Sub Campus Toba Tek Sing, Faisalabad, Pakistan; 5 Department of Entomology, Faculty of Agriculture and Entomology, Islamia University of Bahawalpur, Bahawalpur, 63100 Pakistan; 6 Department of Animal and Poultry Production, Faculty of Agriculture, South Valley University, Qena, 83523 Egypt; 7 Poultry Center, Cooperative Agricultural Research Center, Prairie View A&M University, Prairie View, TX 77446 USA

**Keywords:** Blood profile, Milk composition, Nutrient retention, Urea molasses mineral blocks, Yaks

## Abstract

**Background:**

The study aimed to investigate the effect of urea molasses mineral blocks (UMMB) on nutrient digestibility, productive performance and blood biochemical profile of indigenous yaks under various feeding systems. A total of sixteen yaks were randomly divided into four groups (n = 4 animal per group) and offered the, following feeding systems: (A) stall feeding, (B), urea molasses mineral block (UMMB) + stall feeding, (C) yard feeding and (D) UMMB + yard feeding. Trial lasted for 40 days.

**Results:**

Results showed that nutrients intake (g) and nutrient digestibility (%) of dry matter (DM), organic matter (OM), crude protein (CP), ether extract (EE) and crude fiber (CF) were significantly higher (*p* < 0.05) in stall and yard feeding groups with UMMB licking. Blood zinc, cobalt, hemoglobin (Hb), red blood cell (RBC), glucose and serum glutamate private transaminase (SGPT) significantly *(p <* 0.05*)* increased in stall and yard feeding with UMMB licking. Milk yield, Ca and monounsaturated fatty acid except milk composition improved significantly *(p <* 0.05*)* in stall and yard feeding groups with UMMB licking.

**Conclusion:**

It was concluded that feeding of UMMB improved utilization of low-quality roughages and best results were obtained from stall and yard feedings with UMMB licking as compared to other groups.

## Background

In northern hilly area of Pakistan (Broghil valley), the yak is mostly dependent on natural pastures for its survival. Thus, its dietary condition varies seasonally as there are no supplementary feeds for them [1]. However, only weak and some pregnant or lactating yaks have access to supplementary feeds besides grazing [2]. Consequently, malnourishment of the yak is more likely to become worse in the future. Due to the irrationality in number of farm animals, grazing system and non-scientific management, yak is in a harsh loop of feeding. Low fat and lose weight in the winter season result in low animal productivity [3]. Yak production is reduced due to unavailability of sufficient fodders during winter season by resulting in poor physical conditions, lower growth and reduced fertility.

Since protein supplements are available at high prices. Therefore, ruminants are greatly fed on crop residues of good quality. Using whole blocks such as urea-molasses mineral blocks have a lot of compensations like easy transportation, reduced risk in utilization, enhanced feed efficiency, increased nutrient digestibility, higher milk production, improved blood biochemical profile and reproductive efficiency in ruminants [4–6]. The present research investigated the impact of urea molasses mineral blocks on productive performance, nutrients digestibility, blood biochemical profile, milk yield and composition of yaks under various, feeding systems.

## Results

### Nutrients intakes of animals

Data on nutrients intake in urea molasses minerals (UMMB) per animal per day are shown in Table 1. Nutrients intake either with (stall feeding + UMMB) or (yard feeding + UMMB) statistically increased (*p <* 0.05) as compared to stall and yard feeding alone. In stall feeding groups, DMI ranged (3011.5 to 3801.4 g), OMI (28.02 to 3012.4 g), CPI (711.4 to 779.4 g) and EEI (282.2 to 294.8 g). Similarly, in yard feeding groups, DMI varied (3472.2 to 3701.4 g), OMI (2301 to 3201.4 g), CPI (631.5 to 781.4 g) and EEI (279.3 to 299.7 g).

### Nutrient digestibility

Results of nutrient digestibility of experimental animals among different feeding groups are presented in Table 2. Nutrient digestibility (%) of animals in urea molasses minerals (UMMB) licking groups (stall feeding + UMMB) and (yard feeding + UMMB) significantly enhanced (*p <* 0.05) as compared to stall and yard feeding alone. In stall feeding and stall feeding plus UMMB groups, DM digestibility increased from 59.41 to 63.32%, OM digestibility from 65.43 to 70.43%, CP digestibility from 67.50 to 72.31% and EE digestibility from 46.42 to 53.51%, respectively. Likewise, in yard feeding and yard feeding plus UMMB groups, DM digestibility increased from 53.44 to 68.46%, organic matter digestibility from 60.34 to 75.42%, crude protein digestibility from 62.53 to 77.73% and ether extract digestibility% from 41.41 to 64.41%, respectively. While nutrient digestibility install and yard feeding systems without UMMB decreased significantly when compared with UMMB feeding.

### Blood biochemical profile

Results on biochemical parameters are presented in Table 3. UMMB feeding install and yard feeding systems have not statistically affected (*p >* 0.05) alkaline phosphatase, packed volume cells, SGOT, WBC and serum proteins. However, glucose, Hb, red blood cells (RBC), serum glutamate pyruvate transaminase (SGPT), Zn and Co varied significantly (*p <* 0.05) in UMMB feeding groups as compared to stall and yard feeding without UMMB. In stall feeding and stall feeding with UMMB groups, glucose increased from 54.78 to 59.03 mg/dL, Hb from 12.52 to 16.63 g/dL, red blood cells (RBC) from 5.20 to 5.90 (10^6^/µl), serum glutamate pyruvate transaminase (SGPT) from 26.56 to 32.26 (IU/L), Zn from 0.93 to 1.41 mg/L, Co from 0.59 to 1.02 mg/L, respectively. Similarly, in yard feeding and yard feeding with UMMB groups, glucose increased from 52.94 to 62.01 mg/dL, HB from 11.67 to 15.58 g/dL, RBCs from 5.12 to.6.01 (10^6^/µl), SGPT from 27.13 to 30.58 IU/L, Zn from 0.87 to 0.98 mg/L and Co from 0.47 to 0.98 mg/L, respectively.

### Milk yield and milk composition

Milk yield (L/day/animal) and milk composition are shown in Table 4. It revealed that milk yield varied significantly (*p <* 0.05) with and without UMMB feeding groups. Mean milk yield of yak install and yard with UMMB licking group ranged from 3.65 to 3.95 (L/day) as compared to 2.45 to 2.66 (L/day) in non-UMMB licking groups. Variable results on milk composition were obtained in the present study. Results showed that provision of UMMB in stall and yard feeding did not vary significantly milk protein, fat, total solids and solid not fat (SNF) contents of the yak. These milk components remained similar in all groups. However, UMMB install and yard feeding significantly (*p <* 0.05) improved Ca level and mono unsaturated fatty acids (MUFA).

## Discussion

In the present study, green pasture was offered *ad libitum* to the animals in stall and yard feeding groups and also in stall and yard feeding groups with UMMB. In UMMB groups either stall feeding or yard feeding, considerable enhancement in the profile of nutrients intake was observed. This could be due to beneficial effect of UMMB as a source of soluble nitrogen and with no unfermentable carbohydrates which may have probably improved the action of cellulolytic bacteria in rumen microflora which caused increased fermentation of roughages and therefore high intake [7, 8]. Better utilization of poor quality forage may be mostly enhanced by UMMB supplementation because of higher degradation activity of rumen microbes, particularly, the cellulolytic microflora [9, 10]. Significance of UMMB feeding to dairy cows during mid-lactation is that it provides 6.5 and 14% ME and CP intake to control diet, respectively. However, we cannot ignore the factors like temperature (low and high range), feeding indoor and grazing on natural pasture regarding nutrients intake from the diet. Moreover, higher passage rate of feed particles at lower temperature is also an important factor that provides more rumen space to be fulfilled by feed [10, 11]. However, on the other hand, the present results are not in line with the results of Hosmani [12] as he didn’t observe any increase in nutrient intake due to UMMB feeding. This could be due to difference in forage consumption as an energy source and limited supply of DM through green forage in both studies.

In the present study, clay was used as a binder at 5% level in the blocks. Using cement as a binder has raised some questions from various nutritionists and researchers about possible negative effects on animals (Sansoucy et al., 1988). Due to presence of heavy metals in cement which make it more toxic to animals and transportation problem to experimental site. We have preferred local clay over cement. The consistency observed in final block ensured an even spread of clay binder in the block and also facilitated improved uniform hardness of the block. The strength or compactness of the block was good with clay, may be due to binding of ingredients closely together. Compactness and hardness of clay based UMMBs had positive impact on animal intake which decreases with block hardness in case of cement based blocks (Herrera et al., 2007). Use of clay binder in blocks was efficient and didn’t exert any impact or threat to health of animal (Preston, 1993; Kayouli, 1994).

Like nutrients intake, similar pattern of nutrients digestibility (%) of yaks in stall and yard feeding groups with UMMB has been observed. The digestibility of dry matter (DM) and organic matter (OM) was significantly (*p <* 0.05) higher in stall and yard feeding groups with UMMB licking as compared to stall and yard feeding alone. The availability of better fermentable energy present in UMMB groups might have enhanced the digestibility of DM and OM in these groups. Our results are agreed with Kumar et al. [13], Pate et al. [14] and Mehra et al. [15], who reported similar pattern of nutrient digestibility. A comparable tendency as in DM and OM was also seen in CPD and CFD. UMMB contains degradable and undegradable (bypass) protein, degradable carbohydrates and some minerals [16] which can assist in fulfilling the nutrients need of rumen microbes and ruminant livestock. The discharge of ammonia over a longer phase of time and its utilization by micro-organisms in the rumen may have been supported by simultaneous energy supply, together with a generally improved dietary energy and protein balance in UMMB supplemented groups. An improved CPD in UMMB licking groups could be associated with out flow rate of rumen contents. This out flow rate can be affected by multiple factors like feed availability, air temperature, feed composition, state of feed (solid or liquid), and size of feed particles.

The blood glucose (mg/dL) is considered an important indicator of the normal physiological functions and welfare of animals. The mean values for serum glucose among the feeding groups ranged from 52.9 to 62.0 (mg/dL). In the present research, significant effect of feeding of UMMB on serum glucose was observed among the various groups. Our results are in agreement with the findings of Tiwari [17] and Jain et al. [18], who also noticed that UMMB supplementation has an increasing effect on blood glucose level. On the other hand, Sankar et al. [19] observed no significant effect of UMMB feeding. Also contradictory results were reported by Kumar et al. [20] and Kerketta et al. [21] on fluctuations in blood glucose level.

Like blood glucose, blood Hb is an indicator of erythrocyte (RBC) normal level and general health status of animals. The Hb values varied from 11.6 to 16.4 g/dL across different feeding groups which are significantly comparable and are within the normal range of physiological range. Hb mean values were greater in UMMB feeding groups as compared to without UMMB feeding groups. In line with our results, significantly (*p <* 0.05) higher Hb concentrations were noticed by Kaneko, [22].

There was significant effect (*p* < 0.05) of UMMB feeding groups on RBC and SGPT. Similar findings were reported by Hossain et al. [23], Kioumarsi et al. [24] and Li et al. [25], who observed significant effect of UMMB on these parameters. Their values were within the normal ranges in the present study, which could be attributed to adequate nutrition through UMMB feeding. However, our results are contradictory to Konwar et al. [26], who found low values of RBC and SGPT in UMMB supplemented groups. The activity of SGPT is an indicator of damage to liver and muscles [27, 28]. In our study, concentration of SGPT was increased in UMMB feeding groups which was in the normal range (26.5 to 32.6 (IU/L), therefore, illustrating no adverse and deteriorating effect on hepatic cells and muscle tissues. Our results on SGPT are in agreement with the results of Tiwari et al. [29] and Kerketta et al. [21], who reported significantly higher value of SGPT in UMMB supplemented groups. However, Cenesiz et al. [30] reported no significant effect of UMMB supplementation on the activity of SGPT in lambs.

**Table 1:** Ingredients composition of urea molasses mineral block fed to yak

Ingredients	%	(g/kg)	(g/3kg)
Molasses	43	430	1290
Urea	07	70	210
Limestone	08	80	240
Clay	05	50	150
DCP^1^	03	30	90
Salt	03	30	90
Wheat bran	31	310	930
Total	100	1000	3000

^1^DCP: di calcium phosphate

**Table 2:** Nutrients intake of indigenous yaks reared under different feeding systems

Items	Group-A^1^	Group-B^2^	Group-C^3^	Group-D^4^	*p-*value
Dry matter intake	3011.51 ± 0.82^c^	3801.4 ± 0.73^b^	3472.2 ± 0.87^d^	3701.4^a^ ± 0.77^ab^	0.010
Organic matter intake	2802.49 ± 0.73^c^	3012.4 ± 0.82^b^	2301.4 ± 0.77^d^	3201.4^a^ ± 0.67^ab^	0.001
Crude protein intake	711.42 ± 0.84^c^	779.38 ± 0.70^b^	631.45 ± 0.83^d^	781.41 ± 0.76^ab^	0.001
Ether extract intake	282.23 ± 0.71^c^	294.88 ± 0.81^ab^	279.25 ± 0.77^d^	299.72 ± 0.73^a^	0.010

^a,b,c^ Means values within a row with different superscripts are significantly different (*p <* 0.05)

^1^Group-A: stall feeding, ^2^Group-B: stall feeding + urea molasses mineral block (UMMB), ^3^Group-C: yard feeding, ^4^Group-D: yard feeding + urea molasses mineral block (UMMB).

Significant concentrations of serum Co and Zn among UMMB feeding groups and non without UMMB feeding groups were observed in the present study. Their concentrations were within the reported range [31], which is an indication that yaks under stall and yard feeding with UMMB licking received adequate dietary minerals from the blocks. Blood hematological parameters are important indication of animal health and nutritional status. Factors such as physiological state, environmental condition, disease and stress are also known to influence blood hematological parameters [32].

The present study clearly indicated that mean milk yield/day was increased by 1.3 lit/day (34.2%). This increase could be due to meeting sufficient nutritional requirement through UMMB licking install and yard feeding of yak. This study was carried out in summer and average temperature in summer does not exceed 15° C. During summer, plenty of green forage is available for yak as compared to winter season when forage is available with short grasses and shruby plants and rough grazing conditions are there, which could also be the reason for higher milk yield boosted by UMMB feeding. The findings of the present study are comparable with Singh and Singh [33], who found an increase of 35.9% in milk yield of buffaloes and Avila [34] reported 21% increase in milk yielded due to UMMB supplementation. Nevertheless, our findings are in contrast with Makkar [35] and Upreti et al. [35], who reported lower milk production (14%) and (17.7%), respectively. No effect of feeding system with and without UMMB was observed on milk composition in the present study. Our results are in line with Gupta et al. [36], who reported no effect of UMMB feeding on milk composition. However, in contrast to present study, Makkar, [35] and Ramesh et al. [37] found significant change in milk fat and SNF.

**Table 3:** Nutrients digestibility (%) of indigenous yaks reared under different feeding systems

Items	Group-A^1^	Group-B^2^	Group-C^3^	Group-D^4^	*p-* value
Dry matter digestibility	59.41 ± 0.91^ C^	63.32 ± 0.71^b^	53.44 ± 0.83^d^	68.46 ± 0.71^a^	0.001
Organic matter digestibility	65.43 ± 0.85^ C^	70.43 ± 0.82^b^	60.34 ± 0.79^d^	75.42 ± 0.78^a^	0.001
Crude protein digestibility	67.50 ± 0.82^ C^	72.31 ± 0.74^b^	62.53 ± 0.81^d^	77.73 ± 0.73^a^	0.010
Crude fiber digestibility	46.44 ± 0.78^ C^	53.51 ± 0.67^b^	41.41 ± 0.77^d^	64.41 ± 0.65^a^	0.001

^a,b,c^ Means values within a row with different superscripts are significantly different (*p <* 0.05)

^1^Group-A: stall feeding, ^2^Group-B: stall feeding + urea molasses mineral block (UMMB), ^3^Group-C: yard feeding, ^4^Group-D: yard feeding + urea molasses mineral block (UMMB).

## Conclusions

Dietary feeding of UMMB improved utilization of low-quality roughages and best results were obtained from stall and yard feedings coupled with UMMB provision as compared to stall and yard feedings alone. Using UMMB in yak feeding should be extended in yak producing areas and accepted by local farmers. Future strategy should be planned extensively on microbial community and their degradation potential in yak rumen.

## Materials and methods

### Location of study

The current experiments were conducted in Broghel National Park (BNP) of Pakistan in collaboration of The University of Agriculture Peshawar with Wild Life Department, Peshawar. Broghel National Park (BNP) is located in District Chitral in the northern area of Pakistan and comprised of Broghil valley and small part of Yarkhun Valley. It is spread over 134,800 ha. and the largest National Park in Khyber Pakhtunkhwa (KP) Province of Pakistan. The boundaries of BNP are extended to south of Broghil valley and is situated between 36° 40´ and 36° 55´ (North latitude) and 73° 52´ and 73° 58´ (East longitudes). Broghil valley is part of Broghil Valley National Park which is located at a distance of 250 km from Chitral city. Its elevation ranged from 3,280 to 4,304 m and is at above 13,000 ft from the sea level. The borders of BNP in the north and west also touch with historical Wakhan corridor of Afghanistan. Livestock mainly yaks contribute by 90% to the household economy in this area. 80% of Broghil valley is covered with snow/glacier. The climate is mostly cold and windy during most of the year and average temperature is ˗4° C.

**Table 4:** Blood biochemical parameters of indigenous yaks reared under different feeding systems

Items	Group-A^4^	Group-B^5^	Group-C^6^	Group-D^7^	*p-*value
ALP (IU/L)	187.46 ± 0.82	186.93 ± 0.76	187.13 ± 0.87	186.54 ± 0.70	0.653
Glucose (mg/dL)	54.78 ± 0.73^b^	59.03 ± 0.85^a^	52.94 ± 0.70^b^	62.04 ± 0.74^a^	0.050
Haemoglobin (g/dL)	12.52 ± 0.87^b^	16.63 ± 0.75^a^	11.67 ± 0.82^b^	15.58 ± 0.69^a^	0.014
PCV^1^ (%)	42.61 ± 0.81	43.14 ± 0.78	41.72 ± 0.84	43.16 ± 0.72	0.540
RBC^2^ (10^6^/µL)	5.20 ± 0.25^b^	5.90 ± 0.19^a^	5.12 ± 0.21^b^	6.01a ± 0.11^a^	0.044
Serum protein(g/dL)	8.70 ± 0.81	9.15 ± 0.78	8.57 ± 0.65	9.37 ± 0.57	0.883
WBC^5^ (10³/µL)	111.89 ± 0.67	12.42 ± 0.73	11.53 ± 0.78	12.13 ± 0.65	0.182
Zinc (mg/L)	0.93 ± 0.07^b^	1.41 ± 0.11^a^	0.87 ± 0.02^b^	1.37 ± 0.09^a^	0.037
**Enzymatic Parameters**					
SGOT^3^ (IU/L)	39.92 ± 0.87	41.10 ± 0.75	40.34 ± 0.89	41.46 ± 0.77	0.261
SGPT^4^ (IU/L)	26.56 ± 0.64^b^	32.26 ± 0.71^a^	27.13 ± 0.81^b^	30.58 ± 0.69^a^	0.040

^a,b^Means values within a row with different superscripts are significantly different (*p <* 0.05)

^1^PCV: packed cell volume. ^2^RBC: red blood cells. ^3^SGOT: serum glutamate oxaloacetate transaminase ^4^SGPT: serum glutamate private transaminase, ^5^WBC: white blood cells. ^4^Group-A: stall feeding, ^5^Group-B: stall feeding + urea molasses mineral block (UMMB), ^6^Group-C: yard feeding, ^7^Group-D: yard feeding + urea molasses mineral block (UMMB).

### Experimental animals and design

The study was conducted in Completely Randomized Block Design (CRBD). A total of 16 yaks with same age and body weight (160 ± 13.2 kg) were selected. Yaks were randomly distributed into four equal groups (*n = 4*). Experimental animals were offered feeds under the following feeding systems: (A) stall feeding, (B) urea molasses minerals block (UMMB) + stall feeding, (C) yard feeding, (D) UMMB + yard feeding. Yaks were grazed on natural pasture in open yard (yard feeding group) and same pasture was cut and offered to them in stall feeding group. In stall feeding plus UMMB group, UMMB was placed for *ad libitum* licking of animals. For yard feeding plus UMMB group, yard was fenced with a steel wire tied with trees on four sides of yard. In this group, UMMB was placed in yard for *ad libitum* licking of animals. Animals in this group were under strict observations and it was almost uniform during whole trial. Animals under all feeding systems had free access to clean drinking water and green pasture. For stall feeding, animals were kept in stall (measuring 150 cm × 80 cm × 70 cm, for length × width × height, respectively). Animals were cared according to Care Guidelines of FAH&VS, UAP (Faculty of Animal Husbandry and Veterinary Sciences, The University of Agriculture, Peshawar. The experiment protocol (UAPAN.2019) was approved by the Animal Ethical Committee of FAH&VS, University of Agriculture Peshawar. The trial lasted for 40 days.

### Preparation of urea molasses minerals blocks

The UMMBs were prepared at the Department of Animal Nutrition, Faculty of Animal Husbandry and Veterinary Sciences, The University of Agriculture, Peshawar, Pakistan. It is a rectangular shaped block with a weight of 3 kg, length 20 cm, width 10 cm, height 6 cm, compression 4% and set time 3 min. In the present study, blocks used were of 3 kg, which costs US$ 0.6. Ingredients composition (g/kg) of urea molasses mineral block (UMMB) is given in Table 5. After mixing the ingredients, the mixture was transferred to small block container along with synthetic film to facilitate the block to be removed from the bucket. After 24 h, the blocks were taken out of the container and were left on wood frame for drying in the sun light for at least 5 days.

### Nutrients digestibility trial

At the end of the experiment, a digestibility trial was conducted for seven days. During trial, daily feed intake was recorded. Faeces from each animal in each feeding system were collected, weighed, and frozen at − 10 °C until further chemical analysis. Faecal samples were pre-dried at 60 °C for 48 h before being analyzed. Faecal and feed samples were analyzed for DM content by oven drying (930.13), ash by incineration (942.04), protein by Kjeldahl (974.11), and ether extract (EE) by Soxhlet fat analysis (954.02), as described by the AOAC [38]. Nutrient digestibility was computed by using the following formula: Digestibility, % = [(t − f)/t] × 100, where *t* is the nutrient intake during the collection period [g] and ƒ is the amount of nutrient excreted in faeces [g].

**Table 5:** Milk yield and milk composition of indigenous yaks reared under different feeding systems

Items	Group-A^1^	Group-B^2^	Group-C^3^	Group-D^4^	*p-*value
Milk yield (L/day)	2.45 ± 0.25^b^	3.65 ± 0.20^a^	2.66 ± 0.13^b^	3.95 ± 0.19^a^	0.002
Protein (%)	5.51 ± 0.25	5.10 ± 0.17	5.37 ± 0.11	5.64 ± 0.21	0.671
Fat (%)	6.09 ± 0.84	5.94 ± 0.70	6.12 ± 0.88	5.97 ± 0.77	0.489
Ca (g)	15.87 ± 0.87^b^	16.98 ± 0.72^a^	16.14 ± 0.81^b^	17.12 ± 0.65^a^	0.013
Total solids (%)	18.50 ± 0.77	17.72 ± 0.87	18.94 ± 0.75	17.77 ± 0.86	0.113
Solid not fat (%)	11.34 ± 0.82	11.15 ± 0.71	10.89 ± 0.80	11.22 ± 0.69	0.744
MUFA^5^ (g)	23.53 ± 0.73^b^	31.41 ± 0.62^a^	21.48 ± 0.88^b^	34.45 ± 0.67^a^	0.003

^a,b^Means values within a row with different superscripts are significantly different (*p <* 0.05)

^1^Group-A: stall feeding, ^2^Group-B: stall feeding + urea molasses mineral block (UMMB), ^3^Group- C: yard feeding, ^4^Group-D: yard feeding + urea molasses mineral block (UMMB), ^5^MUFA: Mono unsaturated fatty acids

### Milk yield and milk composition

Yaks were milked twice daily at 04:00 and 16:00 h. Milk yield was recorded daily throughout the experiment. All the hygienic SOPs were followed during the milking time to avoid impurities. Both morning and evening milking of each experimental animal in each group were pooled at ratio of 1:1 to obtain representative milk sample. Representative milk samples were stored at 4 °C for further analysis of milk composition (milk protein, fat, total solids and solid not fat (SNF) using Elko Milk Analyzer Machine according to the method of AOAC [39]. Atomic Absorption Spectrometer (Atomic Spectrophotormetry Analyst 800, Perkin Elmer, Vernon Hills, IL, USA) was used to analyze the contents of calcium and zinc in milk according to the standard method of GB 5413.

### Blood biochemical profile assay

At the end of experiment (40th day), 3 h after the morning feeding, the blood samples from all the experimental yaks were obtained from the jugular vein puncture using 21-guage (1 ½) vacutainer needle (Becton, Dickinson and Company.©, England) into 10 ml blood tube using K3 ethylene diamine tetra acetic acid (EDTA) (BD. Vacutainer®, US) as an anticoagulant. Then blood was centrifuged at 3,500×g for 15 min. at 4 °C for serum collection. The serum samples are transported to laboratory directly and stored at − 20 ° C for further analyses such as blood zinc, cobalt, hemoglobin (Hb), red blood cells (RBC), glucose, serum glutamate private transaminase (SGPT), alkaline phosphate (ALP), cholesterol content, packed cell volume (PCV), white blood cells (WBC), serum glutamate oxaloacetate transaminase (SGOT) and total serum proteins according to standard protocol using commercial diagnostic kit.

### Statistical analysis

The data were analyzed in SPSS.25.0 (2017) [40] using the ANOVA and Completely Randomized Block Design (CRBD). The significant differences between the treatments were confirmed by Tukey as a post-hoc test and distinguished by Duncan’s multiple range [40] as letters on bars (a, b, c). All values were expressed as mean ± SE. The significance differences between feeding groups were considered at *p* < 0.05.

## Data Availability

The datasets used and/or analysed during the current study available from the corresponding author on reasonable request.
